# Overexpression of bacterial *katE* gene improves the resistance of modified tomato plant against *Fusarium oxysporum* f. sp. *lycopersici*

**DOI:** 10.1080/21645698.2021.1903374

**Published:** 2021-03-30

**Authors:** Reda E.A. Moghaieb, Dalia S. Ahmed, Ahmed Gaber, Abdelhadi A. Abdelhadi

**Affiliations:** a Department of Genetics, Faculty of Agriculture Cairo University, Giza, Egypt; b Department of Biology, College of Science, Taif University, Saudi Arabia

**Keywords:** Tomato, hypocotyl, *katE* gene, *Fusarium*, *Agrobacterium*, modified

## Abstract

Tomato (*Solanum lycopersicum* L.) yield is severely affected by *Fusarium* fungal disease. To improve the resistance of tomato against Fusarium oxysporum f. sp. lycopersici (FOL), Escherichia coli katE gene was transformed into two tomato cultivars, namely Castle Rock and Super strain B, via *Agrobacterium tumefaciens*; the transformation efficiency was 5.6% and 3.5%, respectively. The integration of the *katE* gene into T_0_, T_1_, and T_2_ transgenic tomato lines was confirmed using PCR. In addition, DNA dot blot technique confirmed the integration of the *katE* gene into T_2_ transgenic tomato lines. The RT-PCR analysis confirmed that the *katE* gene could be expressed normally in the T_2_ modified lines. Under artificial infection with FOL, the non-modified plants exhibited more severe fungal disease symptoms than those observed in *katE* overexpression (OE) lines. Our analysis showed that the levels of three defense enzymes, namely superoxide dismutase (SOD), catalase (CAT), and peroxidase (POD), were increased during transgenic T_2_ generation pre-treated with FOL. The bioassay of modified lines revealed that an average of 52.56% of the modified Castle Rock cultivar and 50.28% of the modified Super Strain B cultivar showed resistance under *Fusarium* infection. These results clearly indicate that the modified tomato plants, in which the *katE* gene was overexpressed, became more resistant to the infection by FOL than the wild-type plants. Our study has proven that the overexpression of the *E. coli katE* gene in the OE lines could be utilized to develop and improve the resistance against fungal diseases in the modified crops.

## Introduction

Tomato, *Solanum lycopersicum* L., is one of the most important vegetable crops globally due to its high nutritional value and the availability of nutrients such as vitamin C, flavonoids, beta-carotene, and lycopene.^1^ As tomatoes contain lycopene, which has anti-cancer and antioxidant activities, the production and consumption of tomatoes have increased considerably in the past few years.^1,2^ It is noticeable that tomatoes are sold in the form of juice, concentrates, sauce, and soup, in addition to being sold as fresh vegetables. Globally, tomato production was predicted to be at about 180 million tons. Africa and Egypt produced 12% and 3.73% of the total global production of tomatoes, respectively.^3^

The tomato crop can undergo severe losses as a result of exposure to biotic stresses, such as infection with insects and fungi, which can cause a decrease in the production, irrespective of whether grown in open fields or greenhouses.^4^ Tomatoes are infected with several fungal pathogens such as *Phytophthora, Alternaria, Rhizoctonia*, and *Fusarium*, which lead to many devastating diseases such as late blights, early blights, root rots, and wilt. Wilt diseases were considered as one of the most severe diseases that affect tomato production.^5^ One of the most dangerous and widespread diseases is *Fusarium* wilt disease caused by the soil-borne fungus *Fusarium oxysporum* f. sp. *lycopersici* (FOL).^6,7^ FOL causes a decrease in the crop yield by approximately 30–40% and this percentage may increase to about 80% under optimum environmental conditions.^8^ The loss depends on the plant growth stage and environmental conditions.^9^ The optimum conditions for the pathogen growth are acidic soil, dry weather, and warm temperature of about 28°C.^10^ The causal agent, FOL, infects the plant roots, passes from the cortex to the stele, enters the xylem vessels, causes vascular wilts, and finally leads to the death of the plant.^11^ Symptoms of the disease include yellowing of leaves, plant wilting, and a decrease in the plant productivity. The pathogen can survive in the soil for as many as 10 years.^12^

To overcome *Fusarium* wilt disease in tomatoes, many methods such as the use of Agricultural practices (such as crop rotation and soil solarization), synthetic fungicides, resistant cultivars, bio-control agents, and modified lines, which are produced by the modern methods of gene transfer in the plant, are employed.^13^ The fungicides harm humans and the surrounding environment. In addition, resistant strains of the causal agent may develop due to continuous use of fungicides.^14^ Breeding programs using resistant varieties are a reliable method to control *Fusarium* wilt disease; however, this type of resistance was not found to be durable.^15^ Further, bio-control agents have been applied for disease control; nonetheless, these agents alone cannot fully control the disease due to changes in the pH and temperature, which affect the efficiency of the biocontrol agents.^14^ In the early 1980s, with advances in molecular plant biology and a better understanding of infection caused by some pathogens, many complex plant pathways were discovered; the immune response genes and various relevant pathways were also identified in the plants.^16^ The gene transfer methods in plants were evaluated for the possibility of incorporating resistant genes from different species to render the plant disease resistant against infection by fungi and bacteria.^17–19^ In order to overcome *Fusarium* wilt, *Medicago sativa defensin (MsDef1)* gene^18^ and three other genes for pathogenesis-related proteins (*glucanase, chitinase*, and *PR1*)^20^ were transformed into tomato plants to generate modified lines. Moreover, to confer resistance against FOL, the *tomato I-3* resistance gene,^21^ *rice chitinase (RCG3)* gene,^22^ and *Agrobacterium rhizogenes rolA* gene^23^ have been inserted into tomato plants to produce the modified lines.

The overexpression of anti-oxidative enzymes in plant cells was carried out to improve the plant tolerance against different biotic and abiotic stresses.^24^ *E. coli* possess two types of catalases, a bifunctional catalase peroxidase (HPI) encoded by the *katG* gene and a monofunctional catalase (HPII) encoded by the *katE* gene.^25^ The catalase HPII is composed of 732 amino acids and shows higher activity of H_2_O_2_ conversion than the plant catalase.^26^ The *katE* gene has been used to provide oxidative stress,^26^ salt,^27^ and drought tolerance.^28^ The *katE* gene was introduced into different plant species including tobacco,^26^ rice,^27^ jute,^29^ and tomato^30^ for improving their defense system. However, the effectiveness of the *katE* gene in improving plant tolerance against fungal diseases, especially wilt diseases, has not yet been elucidated. Therefore, this study attempted to improve the fungal resistance in tomatoes by overexpressing the bacterial *katE* gene using *Agrobacterium*-mediated technique.

## Results

### *Overexpression of* kat-E *Gene in Tomato*

**Figure 1.**
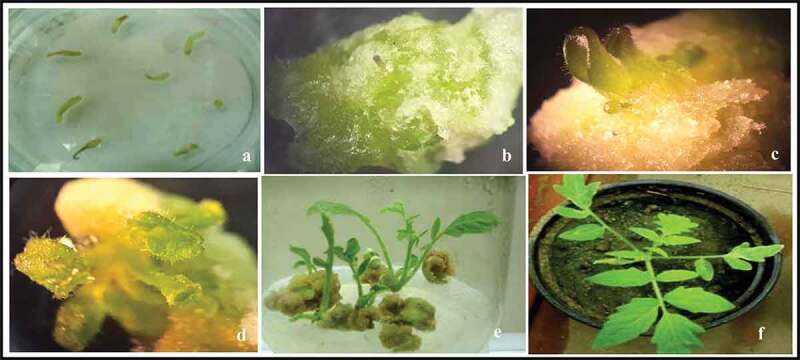
Recovery of fertile modified tomato plants expressing the bacterial *katE* gene. (a) The hypocotyl. (b and c) The callus induction. (d and e) The shoot induction. (f) The modified plants transferred to soil

Two tomato (*Solanum lycopersicum* L.) cultivars, Castle Rock and Super Strain B, were selected for transformation experiments. The hypocotyls (the tomato explants) were co-cultivated with *A. tumefaciens* LBA-4404 harboring the binary vector pBI121-*katE*. The putative transgenic calli were transferred within 3 to 4 weeks to the shoot induction medium. The putative shoots for both the cultivars appeared within 10–15 days. The plantlets that could survive in a medium containing kanamycin sulfate were selected, transferred to plastic pots, and were kept under growth chamber conditions (Figure. 1). The T_0_ and T_1_ seeds were collected, screened by PCR using *katE* specific primers, and planted under suitable conditions; the T_2_ seeds obtained were used for further experiments (supplemented 1).

## Molecular Analysis of Putative *katE* Overexpression (OE) Lines

**Figure 2.**
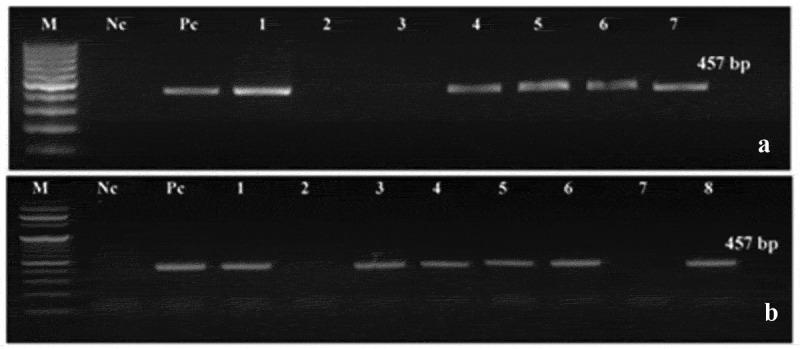
Detection of *katE* gene by PCR in the T_0_ of putative modified plantlets. M: DNA Ladder (100 bp DNA Ladder RTU GeneDirex & TriDye™ 100 bp DNA Ladder BioLabs), Nc: negative control (wild type or non-modified plantlets), Pc: positive control (pBI121-*katE* vector). (a) *katE* gene detection in Castle Rock cultivar; lanes 1 and 4–7: modified plantlets; lanes 2 and 3: non-modified plantlets. (b) *katE* gene detection in Super Strain B cultivar; lanes 1, 3–6, and 8: modified plantlets; lanes 2 and 7: non-modified plantlets

To examine the stable integration of the T-DNA in the putative modified plantlet genomes, the genomic DNA of the T_0_ plantlet was isolated and analyzed by PCR reaction using specific primers to screen the bacterial catalase (*katE*) and *nptII* genes. Clear bands with the expected molecular size of the *katE and nptII* genes (457 and 254 bp, respectively) were detected only in the modified plants; no such bands were seen in the non-modified (control) plants under identical conditions (Figure. 2). Moreover, the transformation efficiency of the two tested tomato cultivars was comparable. A total of 1000 explants were used for each cultivar. For the cultivar Castle Rock, 39 modified plantlets out of 700 regenerated plantlets showed positive results with the PCR analysis and survived on kanamycin sulfate containing medium, thus representing a transformation efficiency of 5.6%. For Super Strain B cultivar, 22 modified plantlets out of 630 regenerated plantlets exhibited positive PCR results, representing a transformation efficiency of 3.5% (Table 1).

**Table 1.** Transformation frequencies of tomato hypocotyl after *Agrobacterium*-mediated transformation using the pBI121-*katE* vector

Cultivars	No. of explants	No. of regenerated shoots	No. of Kana surviving plantlets	katE positive Plantlets (PCR)	Transformation frequency %
Castle Rock	1000	700	39	39	5.6
Super Strain B	1000	630	22	22	3.5

**Figure 3.**
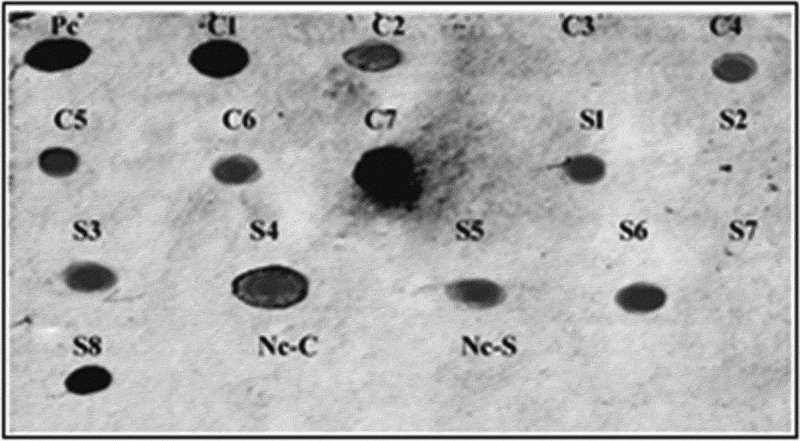
Dot blot analysis with *katE* gene-specific probe for confirming the inheritance of the *katE* gene into T_2_ tomato lines. Nc-C: negative control (non-modified plant) of Castle Rock cultivar, Nc-S: negative control (non-modified plant) of Super Strain B cultivar, Pc: positive control (pBI121-*KatE* vector), C1, C2, and C4-C7: modified lines of Castle Rock cultivar, C3: non-modified line of Castle Rock cultivar, S1, S3-S6, and S8: modified lines of Super Strain B cultivar, S2 and S7: non-modified lines of Super Strain B cultivar

The PCR was used for the screening of T_1_ modified plants using *katE* specific primers. Furthermore, the stable inheritance of the *katE* gene in randomly selected T_2_ modified tomato plants was confirmed using the dot blot technique. Several modified plants from Castle Rock (four lines: 4, 5, 6, and 7) and Super Strain B (four lines: 3, 4, 5, and 6) cultivars exhibited positive results that confirmed that the plants were harboring *katE* gene (Figure. 3). The T_2_ seeds from three positive lines of each cultivar were selected and grown under greenhouse conditions and then were subjected to fungal stress to test for improvement in their fungal resistance.

## Evaluation of the resistance of modified tomato plants against *Fusarium* disease

**Figure 4.**
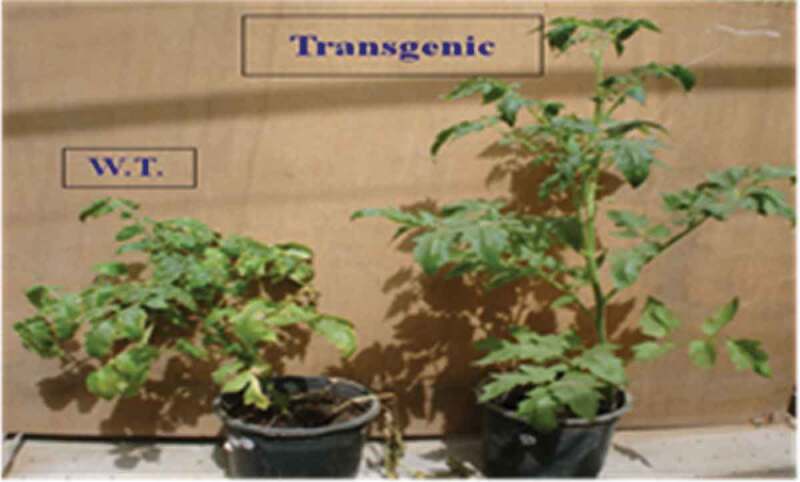
Plant growth of non-modified (wild-type) and *katE* overexpression (modified) plants under artificial infestation with *Fusarium oxysporum* f. sp. *lycopersici.*

The virulent FOL isolate was used to infect modified tomato lines and their corresponding wild type (control) plants. To confirm the infection, the extracted DNA from tomato (wild-type and modified) plants post 1 week of infection with FOL was analyzed by PCR using ITS primers. The data indicated that the PCR product (~550 bp) corroborating with the expected size of the *Fusarium* ITS region was detected only in tomato plants that were previously infected by FOL and this band was not detected in the non-infected plants (data not shown). After 45 days post inoculation, the non-modified (wild type or control) plants exhibited more severe disease symptoms than those observed in the *katE* OE lines (Figure. 4).

**Figure 5.**
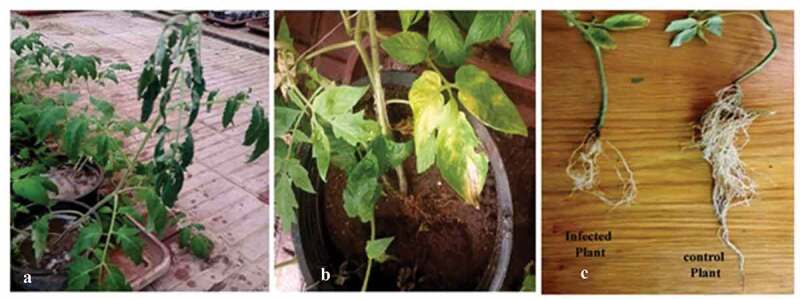
Effect of FOL infection on modified line leaves. (a) non-modified leaves. (b) and roots. (c) of Castle Rock cultivar

The non-modified infected plants showed typical disease symptoms including stunting, yellowing, and premature loss of cotyledons and lower leaves (Figure. 4). Advanced symptoms like pronounced brown lesions that girdle the hypocotyl (root/shoot junction), root rot, wilting, and death were also observed in the non-modified plants, whereas, the *katE* OE lines showed normal growth (Figure. 5).

## Detection of mRNA of *katE* in T_2_ modified tomato plants

**Figure 6.**
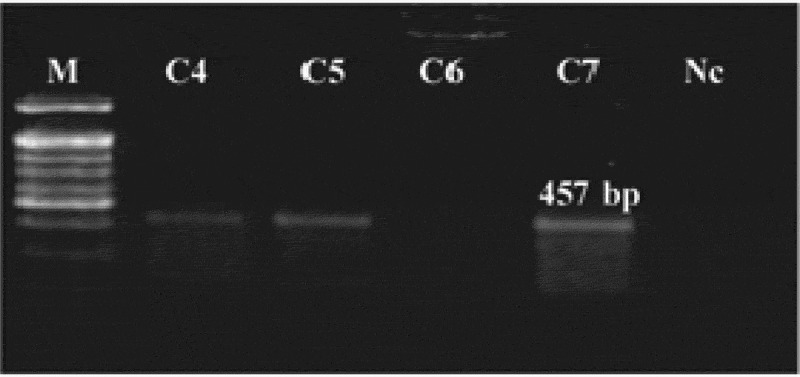
RT-PCR analysis of the *katE* gene mRNA in T_2_ modified tomato lines. C4, C5, and C7: modified lines no. 4–7; Nc: non-modified plant; M: 100 bp DNA ladder

RT-PCR analysis was performed using RNA samples isolated from the T_2_ modified lines produced from the selected T_0_ modified lines no. 4, 5, 6, and 7 of Castle Rock cultivar; these lines showed a positive result upon PCR and dot blot analyses, and the total RNA of the non-modified plant was also isolated. The RT-PCR analysis of the tested lines showed that the mRNA of the *katE* gene in the modified tomato lines 4, 5, and 7 was overexpressed; however, the *katE* gene mRNA in line 6 was not found to be expressed. Furthermore, the mRNA was not detected in the non-modified plants upon RT-PCR analysis (Figure. 6). In addition, the total RNA was also isolated from transgenic line numbers 3, 4, 5, and 6 of Super Strain B cultivar that showed positive results upon PCR and dot blot analyses and was subjected to the RT-PCR analysis. Only three lines, line numbers 3, 4, and 5, gave positive results, while line number 6 did not show any expected band.

## Changes in plant defense enzyme activities under fungal infection

**Figure 7.**
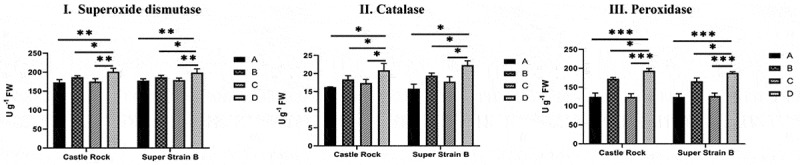
Defense enzyme activities in Castle Rock and Super Strain B cultivars. **I**. SOD activity, **II**. Catalase activity, **III**. Peroxidase activity, **A**: wild type (non-modified) plants, **B**: wild-type plants infected with *Fusarium oxysporum* f. sp. *lycopersici*, **C**: T_2_ modified plants, **D**: T_2_ modified plants infected with *Fusarium oxysporum* f. sp. *lycopersici*. Statistical analysis was performed using GraphPad Prism 8, using a two-way ANOVA with Dunnett’s multiple-comparison posttest; * = *P* < .05, ** = *P* < .01, *** = *P* < .001, **^ns^** = non-significant

The data showed an increase in all enzyme levels under *Fusarium* infection for both the cultivars when compared with the non-infected plant (wild type and modified, Figure. 7). Likewise, the modified plants from each cultivar pre-treated with *Fusarium oxysporum* f. sp. *Lycopersici* showed the highest levels for the three defense enzymes, i.e., superoxide dismutase (SOD), catalase (CAT), and peroxidase (POD). Under natural conditions, all non-infected plants displayed the same pattern in both Castle Rock and Super Strain B cultivars. However, statistical analysis showed that the enzyme activities of SOD, CAT, and POD were highly elevated in infected-modified plants; an increase in the activity of about 1.1-, 1.3-, and 1.5-fold was identified, respectively (Table 2). These results indicate that the expression of the *katE* gene in the modified tomatoes leads to an increase in the concentrations of the three defense-related enzymes that could help in improving the plant’s resistance toward fungal disease.

**Table 2.** Combined analysis of variance of a split-plot design for superoxide dismutase (SOD), catalase (CAT), and peroxidase (POD) enzymes

Source of variation	d.f	SOD	CAT	POD
Replication	2	106.3	4.8	46.6
Factor A (Cultivars)	1	11.48 ^ns^	2.19 ^ns^	48.07 ^ns^
Factor B (Treatment)	3	773.7**	33.9**	6523.2**
AxB	3	16.2	1.05	27.08
Error	14	41.27	0.94	56.74

* and ** indicate significance at 0.05 and 0.01 levels of probability, respectively. ns indicates non-significant value.

## Bioassay for modified plants

The T_1_ seeds of *katE*- modified lines were grown until the T_2_ seeds were obtained, which were then planted to assess the effect of fungal infection. For each cultivar, three individual lines were selected according to PCR, dot blot, and RT-PCR analyses to carry out the bioassay (seeds produced from T_0_ modified lines no. 4, 5, and 7 for Castle Rock cultivar and line numbers 3, 4, and 5 for Super Strain B cultivar). The modified and non-modified plants (26 seedlings) were infested with *Fusarium oxysporum* f. sp. *lycopersici*. In Castle Rock and Super Strain B cultivars, the data revealed that the average number of symptomless (resistance) of all modified line plants under *Fusarium* infection was 52.56% and 50.28%, respectively (Table 3). In both the cultivars, the control (non-modified) plants showed severe wilt symptoms. However, the modified plants that were resistant to *Fusarium* showed normal morphology, fertility, and ability to produce natural seeds. These results indicate that the expression of the *katE* gene in the modified tomato plants improves their resistance toward fungal disease.

**Table 3.** Percentage of susceptible plants, plants showing mild symptoms, and resistant plants in modified and non-modified tomato lines after the *Fusarium oxysporum* f. sp. *lycopersici* infection

	Castle Rock				Super Strain B		
	% symptomless	% mild symptoms	% Susceptible		% symptomless	% mild symptoms	% Susceptible
Modified line 4	53.84	19.23	26.92	Modified line 3	50.0	19.23	30.76
Modified line 5	46.15	26.92	26.92	Modified line 4	53.84	15.38	30.76
Modified line 7	57.69	11.53	30.76	Modified line 5	50.0	15.38	34.61
Average	52.56	19.2	28.2	Average	50.28	16.66	32.04
Non-modified (Control)	11.53	15.38	73.07	Non-modified (Control)	19.23	26.92	53.84

## Discussion

Throughout their life cycle, plants are subjected to different biotic stresses including insect, bacterial, and fungal infections. *Fusarium oxysporum* f. sp. *lycopersici (Sacc.)* is the main causative agent of tomato wilts disease. It is considered one of the most destructive tomato diseases and has caused major losses in tomato production worldwide. The tomato cultivars vary in their resistance toward *Fusarium* according to their genetic makeup. *Agrobacterium* transformation with *katE* gene was utilized in this study to increase the resistance of two tomato cultivars against *Fusarium* infection. Many reports have demonstrated the wide use of transformation techniques to improve the resistance of tomato cultivars against *Fusarium*. The Castle Rock cultivar of tomato was developed by *MsDef1* gene overexpression to improve the FOL resistance.^18^ In their study, the authors reported that the modified lines became more resistant to the FOL pathogen. The wheat chitinase gene (*chi194*) encoding a 33-kDa chitinase protein was overexpressed in tomato plants (cv. Pusa Ruby). The transgenic tomato lines with high chitinase activity were found to be highly resistant to the fungal pathogen *Fusarium oxysporum* f. sp. *lycopersici*.^31^ The pathogen resistance was also enhanced in tomato plants transformed by the *Agrobacterium*-mediated technique using PBI121-ChiGluPRl plasmid containing pathogenesis-related protein genes.^20^ The resistance genes *I, I-2*, and *I-3* have been incorporated into cultivated tomato plants (*Solanum lycopersicum*) from wild tomato species to confer resistance against *Fusarium oxysporum* f. sp. *lycoperici* races 1, 2 and 3, respectively.^21^ Besides, the expression of tobacco osmotin, bean chitinase,^32^ and rice chitinase^33^ genes was utilized to produce tomato transgenic lines resistant against *Fusarium oxysporum* f. sp. *lycopersici*. Bettini *et al*.^23^ transformed the tomato plants with *Agrobacterium rhizogenes rolA* gene to evaluate the role of this gene in the defense response of plants to FOL. Bacterial *katE* gene has been introduced into many different plants to evaluate its role in improving plant response under several stress conditions; examples include transgenic canola (*Brassica napus L*) for resistance against the airborne pathogenic fungi,^34^ transgenic tobacco for drought and heavy light tolerance^28^ and transgenic rice for salt tolerance improvement.^27,35^

Limones *et al*.^36^ indicated the involvement of ROS in chickpea fusarium wilt caused by *Fusarium oxysporum* f. sp. *ciceris*. Their results indicated that infection by *Fusarium oxysporum* f. sp. *ciceris* led to substantial changes in the antioxidant status of chickpea. They concluded that the induction of antioxidant enzymes during chickpea fusarium wilt suggests that changes in oxidative metabolism may be a common plant defense response. Catalase acts as an effective ROS scavenger to avoid oxidative damage. Su *et al*.^37^ have suggested that catalase activity may have a positive correlation with smut resistance in sugarcane. They reported that the enzymatic activity of catalase was higher in Yacheng 05–179 (resistant) variety than in Liucheng 03–182 (susceptible) variety. Catalase of *E. coli* cells (*katE*) is one of the scavenging enzymes that responds to H_2_O_2_ degradation and is involved in the resistance to oxidative stress that is induced under different stresses in many organisms.^27,29^ It is well known that H_2_O_2_ is considered as one of the most important ROS formed and is known to inhibit the plant growth. It also acts as a diffusible signal that induces downstream defense proteins.^38^ In the present study, modified tomato plants expressing the bacterial *katE* gene were obtained. Modified and wild-type tomato plants were artificially infested with *Fusarium oxysporum*. The results showed that overexpression of the *katE* gene significantly improved the antifungal resistance in the modified lines. Moreover, the activity of SOD, CAT, and POD enzymes showed a significant increase in the modified tomato lines as compared to that in the non-modified plants (control) under fungal infestation. Manikandan and Raguchander^39^ increased the *Fusarium* defensive response in tomatoes using a liquid formulation of *Pseudomonas fluorescens* (Pf1). This led to an increase in defense mechanism of enzymes such as SOD, CAT and POD. This was in agreement with our results, which showed that an increase in the activities of SOD, CAT, and POD enzymes could lead to enhanced defensive responses in plants against *Fusarium*. Furthermore, Mandal *et al*.^40^ have investigated the responses of tomato plants infected with *Fusarium oxyspor*um f. sp. *lycoperici* by estimating the activity of the antioxidant enzymes. They found that the activities of the antioxidative enzymes, such as SOD, CAT, and POD, increased in response to pathogen inoculation. In addition, El-Awady *et al*.,^34^ using chemical analyses, found high levels of catalase and peroxidase enzymes in the modified canola plants. These results agree with the present data indicating that *Fusarium*-infected plants tend to accumulate a much higher concentration of the detoxifying enzymes, especially CAT, to resist the fungal infection.

As a result of the expression of the bacterial *katE* gene in tomato plants, the resistance toward *Fusarium* may improve via the plant’s enhanced ability to remove H_2_O_2_. Although H_2_O_2_ is essential for signaling related to pathogen invasion and defense, the accumulation of excessive H_2_O_2_ results in oxidative stress that can damage the plant cells.^41^ The breakdown of H_2_O_2_ prevents the formation of the highly toxic hydroxyl radical (ׄ.OH) in the plant cells.^42^ Therefore, we envisage that the expression of the *katE* gene in tomato plant cells might have an important role in the enzymatic H_2_O_2_ scavenging mechanism. This enzymatic defense mechanism involves SOD and CAT enzymes.^43^ The main role of SOD is to rapidly convert .OH to H_2_O_2_ and the produced H_2_O_2_ is then converted to oxygen and water by CAT and POD.^44,45^ Finally, catalases and other antioxidant enzymes play a key role in defending plants from the harm caused by ROS.^46^ They maintain the normal balance of ROS in the cells; enhanced ROS production leads to gene mutations, lipid peroxidation, and disruption of molecule building and indirectly influences almost every cell activity, ultimately leading to plant cell death.^47,48^

## Conclusion

The integration of the *katE* gene into the tomato genome was confirmed using various molecular methods. The modified tomato plants with the *katE* gene showed resistance to FOL by increasing the cellular activity of defense enzymes such as CAT, SOD, and POD. Their increased cellular levels improved plant resistance against *Fusarium* wilt by eliminating damage caused by excessive H_2_O_2_. Based on these results, it can be suggested that the expression of the *katE* gene may protect other plants from oxidative damage under fungal infection as well. The results of this study have immense importance in the field of agricultural production as they would facilitate the production of modified tomato cultivars that are more resistant to *Fusarium* wilt disease, which causes huge losses in the yield. These resistant cultivars would also be included in various breeding programs to improve tomato production.

## Materials and methods

### Plant materials

Two tomato (*Solanum lycopersicum* L.) cultivars, Super Strain B and Castle Rock, were used in all treatments. The cultivars were kindly provided by Vegetable Crops Department, Faculty of Agriculture, Cairo University, Egypt. The seeds and seedlings were prepared and maintained as described by Manikandan and Raguchander.^39^

### Primers

For *Fusarium* detection and identification on infected plants, the internal transcribed spacer (ITS) region was amplified using primers ITS1 5ʹ-TCCGTAGGTGAACCTGCGG-3ʹ and ITS4 5ʹ-TCCTCCGCTTATTGATATGC-3ʹ.^49^ The *katE* gene was detected in modified tomato plants using two sets of oligonucleotide primers specific for the *nptII* and *katE* genes (Table 4).

**Table 4.** The nucleotide sequence of the *katE* and *nptII* primers used for PCR analysis

Genes	Sequences	Expected size (bp)	Referance
***katE***	FkatE. 5ʹ-AAAAACTCACCGGACGTGAC-3ʹ RkatE. 5ʹ-TAATTCGCCGGGTTAGTGTC-3’	457	^50^
***nptII***	FnptII. 5ʹ- CGCAGGTTCTCCGGCCGCTTGGGTGG-3ʹ RnptII. 5ʹ- GCAGCCAGTCCCTTCCCGCTTCAG-3’	254	

## Fungal pathogen preparation

The fungal artificial infection was done by highly virulent isolate of FOL. Potato Dextrose Agar (PDA) medium was used to maintain the FOL that was received from the Plant Pathology Department, Faculty of Agriculture, Cairo University. Fusarium suspensions were prepared as described by Manzo *et al*. ^51^ Tomato seedlings were exposed to fungal infection as explained by Manikandan and Raguchander.^39^

## Transformation and regeneration conditions

**Figure 8.**
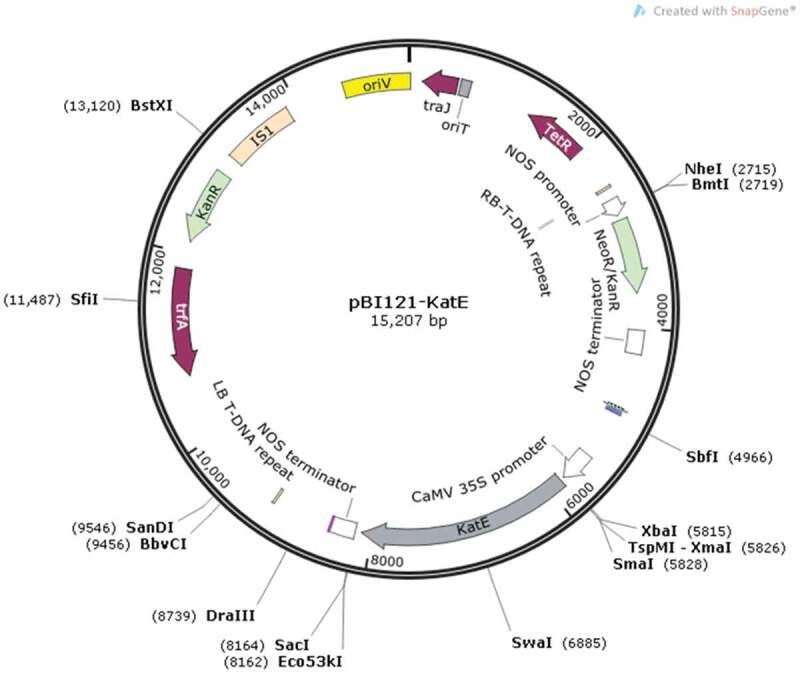
Schematic illustration of the pBI121-*KatE* constructed binary vector; Abbreviations: RB: right border; nosP: nopaline synthase (NOS) promoter; *NPT-II* (Kan^R^): *neomycin phosphotransferase-II* gene; nosT: nopaline synthase (NOS) terminator; 35SP: cauliflower mosaic virus (CaMV) 35S promoter; *katE: E. coli catalase* gene; LB: left border

To produce genetically modified tomato plants, *Agrobacterium tumefaciens* LBA4404 was used in the process of plant genetic transformation using the pBI121-*katE* constructed binary vector, which was kindly provided by Prof. Shigeru Shigeoka, Kinki University, Japan. The T-DNA region contains the right border and expression cassettes including the neomycin phosphotransferase II (NPTII) selection marker under regulatory of nopaline synthase (nos) promoter and nopaline synthase (nosT) terminator, *E. coli* catalase gene (*katE*) under control of cauliflower mosaic virus (CaMV) 35S promoter and nopaline synthase (nosT) terminator, and the left border (Figure 8).

Free hormones MS medium was utilized for Castle Rock and Super strain B seeds germinated. After germination, the hypocotyl was used as an explant for transformation procedure. The pBI121-*katE* vector was transformed into *A. tumefaciens* strains LBA4404 using the heat shock method as described previously.^52^ T_0_ plantlets were transferred to pots and grown to maturity in a greenhouse. T_1_ seeds were collected and germinated in an MS medium containing 50 mg/l kanamycin. The germinated seedlings were transferred to a greenhouse and even used to get T_2_ seeds that were planted and used for further analysis.^52^

## Extraction and purification of genomic DNA

The mass of FOL mycelium was used to extract the fungal genomic DNA according the manufacturer's protocol of DNeasy® Plant Mini Kit (QIAGEN, cat. No. 69104, USA). Total plant DNA was extracted from modified and wild-type tomato plants according to the method of Rogers and Bendich.^53^

## PCR analysis

The ITS and *katE* PCRs were done using the OnePCR^Tm^ Kit (GeneDirex, cat. No. MB203-0100, Taiwan) in a 20 μl total volume consisting of template DNA (5 ng), each primer (0.4 μM), 10 μl of OnePCR pre-mixed solution (2X) and ddH_2_O to 20 μl total volume. The reaction program was achieved as follows: 94°C for 5 min, followed by 35 cycles of 95°C for 45 s, 52°C for 1 min for ITS region and 56°C for 1 min for *katE* gene, 72°C for 90 s and finally 72°C of 10 min.

## DNA Dot blot analysis

DNA was extracted from both modified and non-modified plants as described by Rogers and Bendich.^53^ The DNA was denatured from both lines at 95°C for 10 min. DNA was then spotted on a nylon membrane according to the standard method.^54^ *KatE* PCR product was labeled and used as a probe. Biotin Chromogenic Detection kit was utilized for both hybridization and detection according to the manufacturer’s instructions (Ferments Life Sciences, cat. No. K0661, K0662, USA).

## Reverse Transcription PCR (RT-PCR)

RT-PCR analysis was carried out to evaluate the *katE* gene expression in modified tomato plants. For the modified and non-modified (control or wild-type) infected plants, total RNA was purified from leaf tissue samples using Total RNA Isolation Kit (GeneDirex, Cat. No. SN020-0100, Taiwan). For cDNA synthesis, the reaction was performed using RevertAid™ First-Strand cDNA Synthesis Kit (Ferments Life Sciences, USA). The specific primer for the *katE* gene that listed in (Table 4) was applied to the RT-PCR reaction. The final RT-PCR product was separated in 1% agarose gels and examined.

## Estimation of enzyme activity

To estimate the enzyme activity as a result of fusarium infection, three different enzymes were estimated in the plant samples which are superoxide dismutase (SOD), catalase (CAT), and peroxidase (POD). Therefore, different plant leaf tissue samples were prepared as described by Chakraborty *et al*.^55^ CAT was determined spectrophotometrically by measuring the decrease in absorbance at 240 nm resulting from the decomposition of H_2_O_2_ according to Aebi.^56^ The reaction mixture was 100 mM sodium phosphate buffer (pH 7.0), 30 mM H_2_O_2_ and 100 µL of crude extract in a total volume of 1 mL. The method described by Onsa *et al*.^57^ was used to determine POD activity. The reaction mixture contained 0.1 ml H2O2, 1.0 ml of 0.03 M guaiacol as a substrate, 1.8 ml of 0.1 M acetate buffer (pH 4.5), and 0.1 ml of enzyme solution. An increase in optical density at 420 nm was recorded to assay the activity. SOD enzyme activity was measured according to the method of Kumar *et al*.^58^ The reaction mixture contained 10 mM EDTA, 50 mM nitroblue tetrazolium (NBT), 10 mM riboflavin, 50 mM sodium phosphate buffer (pH 7.6), 50 mM sodium carbonate, 12 mM L-methionine, and [100μL][100μL][100μL][100μL] crude extract in a final volume of 1 mL. The reaction was started by adding riboflavin, which started the light-mediated reaction. Tubes were incubated at room temperature for 25 min under white light. Next, the reaction was measured at 560 nm using a spectrophotometer. One unit of enzyme activity between CAT and POD is defined as the amount of the enzyme required for reducing 1μmol of the substrate per min while, in SOD, one unit is defined as the amount of the enzyme that causing 50% inhibition of the substrate. The enzyme activities were done on four different levels (four treatments); (1) wild type (non-modified) plants, (2) wild-type plants infected with FOL, (3) T_2_ transgenic plants, and (4) T_2_ modified plants infected with the fungus.

## Disease resistance analysis

The analysis was carried out as described by Abdallah *et al*.^18^ The resistance against *Fusarium* fungus was scored in the wild type (non-modified or control), T_2_ of modified plants. The severity of the wilt symptoms was examined by calculating the percentage of symptomless (resistant), mild symptoms (tolerant) and susceptible plants. The symptoms and infection severity assayed 45 days after the infestation.

## Statistical analysis

The test of normality distribution was done according to Shapiro and Wilk^59^ method by using SPSS v. 17.0 (2008) computer package. A randomized complete block design with two factors was used to analyze all data with three replications for each parameter. The one-way analysis of variance (ANOVA) was carried out using Graph Pad Prism 8 for Windows 10 computer software package on one factor (treatments) Dunnett’s multiple comparisons test at α = 0.05.

## References

[1] Raiola A, Rigano MM, Calafiore R, Frusciante L, Barone A. Enhancing the health-promoting effects of tomato fruit for biofortified food. Mediators of Inflammation. 2014;12(3):345–350. doi:10.1155/2014/139873. PMC3972926 24744504

[2] Wu X, Zhu W, Zhang H, Ding H, Zhang HJ. Exogenous nitric oxide protects against salt-induced oxidative stress in the leaves from two genotypes of tomato (*Lycopersicom esculentum Mill*.). Acta Physiologiae Plantarum. 2011;33(4):1199–1209. doi:10.1007/s11738-010-0648-x.

[3] FAOSTAT. Food and Agriculture Organization of the United Nations. Rome: FAO; 2019.

[4] Alatar AA, Faisal M, Abdel-Salam EM, Canto T, Saquib Q, Javed SB, El-Sheikh MA, Al-Khedhairy AA. Efficient and reproducible in vitro regeneration of *Solanum lycopersicum* and assessment genetic uniformity using flow cytometry and SPAR methods. Saudi J Biol Sci. 2017;24(6):1430–1436. doi:10.1016/j.sjbs.2017.03.008. 28855842 PMC5562467

[5] Gleason ML, Edmunds BA. Tomato diseases and disorders. Iowa (USA): Iowa State University, Ames; 2006.

[6] Reis A, Costa H, Boiteux LS, Lopes CA. First report of *Fusarium oxysporum* f. sp. *lycopersici race* 3 on tomato in Brazil. Fitopatologia Brasileira. 2005;30(4):426–428. doi:10.1590/S0100-41582005000400017.

[7] Sudhamoy M, Mallick N, Mitra A. Salicylic acid-induced resistance to *Fusarium oxysporum f. sp. lycopersici* in tomato. Plant Physiology and Biochemistry. 2009;47(7):642–649. doi:10.1016/j.plaphy.2009.03.001. 19329332

[8] Nirmaladevi D, Venkataramana M, Srivastava RK, Uppalapati SR, Gupta VK, Yli-Mattila T, Tsui KMC, Srinivas C, Niranjana SR, Chandra NS. Molecular phylogeny, pathogenicity and toxigenicity of *Fusarium oxysporum* f. sp. *lycopersici*. Sci Rep. 2016;6:21367. doi:10.1038/srep21367. 26883288 PMC4756691

[9] Srinivas C, Devi DN, Murthy KN, Mohan CD, Lakshmeesha TR, Singh B, Kalagatur NK, Niranjana SR, Hashem A, Alqarawi AA, et al. *Fusarium oxysporum* f. sp. *lycopersici* causal agent of vascular wilt disease of tomato: biology to diversity– a review. Saudi Journal of Biological Sciences. 2019;26(7):1315–1324. doi:10.1016/j.sjbs.2019.06.002. 31762590 PMC6864208

[10] Debbi A, Boureghda H, Monte E, Hermosa R. Distribution and genetic variability of *Fusarium oxysporum* associated with tomato diseases in Algeria and a biocontrol strategy with indigenous *Trichoderma* spp. Front. Microbiol. 2018;9:282. doi:10.3389/fmicb.2018.00282. 29515557 PMC5826367

[11] Bowers JH, Locke JC. Effect of botanical extracts on the population density of Fusarium oxysporum in soil and control of Fusarium wilt in the greenhouse. Plant Dis. 2000;84(3):300–305. doi:10.1094/PDIS.2000.84.3.300. 30841245

[12] Akbar A, Hussain S, Ullah K, Fahim M, Ali GS. Detection, virulence and genetic diversity of Fusarium species infecting tomato in Northern Pakistan. PLoS ONE. 2018;13(9):e0203613. doi:10.1371/journal.pone.0203613. 30235252 PMC6147440

[13] Hassan HA. Biology and integrated control of tomato wilt caused by *Fusarium oxysporum lycopersici*: a comprehensive review under the light of recent advancements. J Bot Res. 2020;3(1):84–99. doi:10.36959/771/564.

[14] Singh R, Biswas SK, Nagar D, Singh J, Singh M, Mishra YK. Sustainable integrated approach for management of Fusarium wilt of tomato caused by *Fusarium oxysporum* f. sp. *lycopersici* (Sacc.) Synder and Hansen. Sustainable Agriculture Research. 2015;4(1):138–147. doi:10.5539/sar.v4n1p138.

[15] Jones JW, Dayan E, Allen LH, Van Keulen H, Challa H. A dynamic tomato growth and yield model (TOMGRO). Transactions of the ASAE. 1991;34(2):663–0672. doi:10.13031/2013.31715.

[16] Rommens CM, Kishore GM. Exploiting the full potential of disease-resistance genes for agricultural use. Curr Opin Biotechnol. 2000;11(2):120–125. doi:10.1016/s0958-1669(00)00083-5. 10753764

[17] Dessoky ES, Ismail RM, Elarabi NI, Abdelhadi AA, Abdallah NA. Improvement of sugarcane for borer resistance using *Agrobacterium* mediated transformation of *cry1Ac* gene. GM Crops & Food. 2021;12(1):47–56. doi:10.1080/21645698.2020.1809318. 32862762 PMC7595610

[18] Abdallah NA, Shah D, Abbas D, Madkour M. Stable integration and expression of a plant *defensin* in tomato confers resistance to fusarium wilt. GM Crops. 2010;1(5):344–350. doi:10.4161/gmcr.1.5.15091. 21844692

[19] Yoshimura S, Yamanouchi U, Katayose Y, Toki S, Wang ZX, Kono I, Kurata N, Yano M, Iwata N, Sasaki T. Expression of *Xa1*, a bacterial blight resistance gene in rice, is induced by bacterial inoculation. Proc Nat Acad Sci. 1998;95:1663–1668. doi:10.1073/pnas.95.4.1663. 9465073 PMC19140

[20] Dolatabadi B, Ranjbar G, Tohidfar M, Dehestani A. Genetic transformation of tomato with three pathogenesis-related protein genes for increased resistance to *Fusarium oxysporum* f.sp. *lycopersici*. J Plant Mol Breed. 2014;2(1):1–11. doi:10.22058/JPMB.2014.8424.

[21] Catanzariti A, Lim GTT, Jones DA. The tomato I-3 gene: a novel gene for resistance to Fusarium wilt disease. New Phytologist. 2015;207(1):106–118. doi:10.1111/nph.13348. 25740416

[22] Jabeen N, Chaudhary Z, Gulfraz M, Rashid H, Mirza B. Expression of *rice chitinase* gene in genetically engineered tomato confers enhanced resistance to fusarium wilt and early blight. Plant Pathol J. 2015;31(3):252–258. doi:10.5423/PPJ.OA.03.2015.0026. 26361473 PMC4564150

[23] Bettini PP, Santangelo E, Baraldi R, Rapparini F, Mosconi P, Crinò P, Mauro ML. Agrobacterium rhizogenes rolA gene promotes tolerance to *Fusarium oxysporum* f. sp. *lycopersici* in transgenic tomato plants (*Solanum lycopersicum* L.). Journal of Plant Biochemistry and Biotechnology. 2016;25(3):225–233. doi:10.1007/s13562-015-0328-4.

[24] Polidoros AN, Mylona PV, Scandalios JG. Transgenic tobacco plants expressing the maize *Cat2* gene have altered catalase levels that affect plant-pathogen interactions and resistance to oxidative stress. Transgenic Research. 2001;10(6):555–569. doi:10.1023/A1013027920444. 11817543

[25] Ossowski IV, Mulvey MR, Leco PA, Borys A, Loewen PC. Nucleotide sequence of *Escherichia coli katE*, which encodes catalase HPII. J Bacteriol. 1991;173(2):514–20. doi:10.1128/jb.173.2.514-520.1991. 1987146 PMC207040

[26] Shikanai T, Takeda T, Yamauchi H, Sano S, Tomizawa K, Yokota A, Shigeoka S. Inhibition of ascorbate peroxidase under oxidative stress in tobacco having bacterial catalase in chloroplasts. FEBS Lett. 1998;428(1–2):47–51. doi:10.1016/S0014-5793(98)00483-9. 9645472

[27] Nagamiya K, Motohashi T, Nakao K, Prodhan SH, Hattori E, Hirose S, Ozawa K, Ohkawa Y, Takabe T, Takabe T, et al. Enhancement of salt tolerance in transgenic rice expressing an *Escherichia coli catalase* gene, *katE*. Plant Biotechnol Rep. 2007;1:49–55. doi:10.1007/s11816-007-0007-6.

[28] Miyagawa Y, Tamoi M, Shigeoka S. Evaluation of the defense system in chloroplasts to photooxidative stress caused by paraquat using transgenic tobacco plants expressing catalase from *Escherichia coli*. Plant Cell Physiol. 2000;41(3):311–320. doi:10.1093/pcp/41.3.311. 10805594

[29] Islam MS, Azam MS, Sharmin S, Sajib A, Alam MM, Reza MS, Ahmed R, Khan H. Improved salt tolerance of jute plants expressing the *katE* gene from *Escherichia coli*. Turk J Biol. 2013;37:206–211. doi:10.3906/biy-1205-52.

[30] Mohamed EA, Iwaki T, Munir I, Tamoi M, Shigeoka S, Wadano A. Overexpression of bacterial catalase in tomato leaf chloroplasts enhances photo-oxidative stress tolerance. Plant Cell and Environment. 2003;26:2037–2046. doi:10.1046/j.0016-8025.2003.01121.x.

[31] Girhepuje PV, Shinde GB. Transgenic tomato plants expressing a wheat endochitinase gene demonstrate enhanced resistance to *Fusarium oxysporum* f. sp. *lycopersici*. Plant Cell Tissue Organ Cult. 2011;105(2):243–251. doi:10.1007/s11240-010-9859-5.

[32] Ouyang B, Chen YH, Li HX, Qian CJ, Huang SL, Ye ZB. Transformation of tomatoes with *osmotin* and *chitinase* genes and their resistance to *Fusarium* wilt. J Hortic Sci Biotechnol. 2005;80(5):517–522. doi:10.1080/14620316.2005.11511971.

[33] Jabeen N, Chaudhary Z, Gulfraz M, Rashid H, Mirza B. Expression of *rice chitinase* gene in genetically engineered tomato confers enhanced resistance to fusarium wilt and early blight. Plant Pathol J. 2015;31(3):252–258. doi:10.5423/PPJ.OA.03.2015.0026. 26361473 PMC4564150

[34] El-Awady M, Moghaieb REA, Haggag W, Youssef SS, El-Sharkawy AM. Transgenic canola plants over-expressing bacterial catalase exhibit enhanced resistance to *Peronospora parasitica* and *Erysiphe polygoni*. Arab J Biotechnol. 2008;11:71–84.

[35] Moriwaki T, Yamamoto Y, Aida T, Funahashi T, Shishido T, Asada M, Prodhan SH, Komamine A, Motohashi T. Overexpression of the *Escherichia coli* catalase gene, *katE*, enhances tolerance to salinity stress in the transgenic indica rice cultivar, BR5. Plant Biotechnol Rep. 2008;2:41–46. doi:10.1007/s11816-008-0046-7.

[36] Limones C, Hervás A, Navas-cortés JA, Jiménez-Díaz RM, Tena M. Induction of an antioxidant enzyme system and other oxidative stress markers associated with compatible and incompatible interactions between chickpea (*Cicer arietinum* L.) and *Fusarium oxysporum* f. sp. *ciceris*.. Physiol Mol Plant Pathol. 2002;61:325–337. doi:10.1006/pmpp.2003.0445.

[37] Su Y, Guo J, Ling H, Chen S, Wang S, Xu L, Que Y. Isolation of a novel peroxisomal *catalase* gene from sugarcane, which is responsive to biotic and abiotic stresses. PloS One. 2014;9(1):e84426. doi:10.1371/journal.pone.0084426. 24392135 PMC3879312

[38] Lamb C, Dixon RA. The oxidative burst in plant disease resistance. Annu Rev Plant Physiol Plant Mol Biol. 1997;48:251–275. doi:10.1146/annurev.arplant.48.1.251. 15012264

[39] Manikandan R, Raguchander T. *Fusarium oxysporum* f. sp. *lycopersici* retardation through induction of defensive response in tomato plants using a liquid formulation of *Pseudomonas fluorescens* (Pf1). Eur J Plant Pathol. 2014;140:469–480. doi:10.1007/s10658-014-0481-y.

[40] Mandal S, Mitra A, Mallick N. Biochemical characterization of oxidative burst during interaction between *Solanum lycopersicum* and *Fusarium oxysporum* f. sp. *lycopersic*i. Physiol Mol Plant Pathol. 2008;72:56–61. doi:10.1016/j.pmpp.2008.04.002.

[41] Laloi C, Apel K, Danon A. Reactive oxygen signalling: the latest news. Curr Opin Plant Biol. 2004;7(3):323–328. doi:10.1016/j.pbi.2004.03.005. 15134754

[42] Huang H, Ullah F, Zhou D, Yi M, Zhao Y. Mechanisms of ROS regulation of plant development and stress responses. Front Plant Sci. 2019;10:800. doi:10.3389/fpls.2019.00800. 31293607 PMC6603150

[43] Apel K, Hir H. Reactive oxygen species: metabolism, oxidative stress, and signal transduction. Annu Rev Plant Biol. 2004;55:373–399. doi:10.1146/annurev.arplant.55.031903.141701. 15377225

[44] Gechev TS, Van Breusegem F, Stone JM, Denev I, Laloi C. Reactive oxygen species as signals that modulate plant stress responses and programmed cell death. Bioessays. 2006;28:1091–1101. doi:10.1002/bies.20493. 17041898

[45] Mittler R. ROS are good. Trends Plant Sci. 2017;22:11–19. doi:10.1016/j.tplants.2016.08.002. 27666517

[46] Foyer CH, Noctor G. Stress-triggered redox signalling: what’s in pROSpect? Plant Cell Environ. 2016;39(5):951–964. doi:10.1111/pce.12621. 26264148

[47] Mittler R, Vanderauwera S, Gollery M, Van Breusegem F. Reactive oxygen gene network of plants. Trends Plant Sci. 2004;9(10):490–498. doi:10.1016/j.tplants.2004.08.009. 15465684

[48] Vellosillo T, Vicente J, Kulasekaran S, Hamberg M, Castresana C. Emerging complexity in reactive oxygen species production and signaling during the response of plants to pathogens. Plant Physiol. 2010;154(2):444–448. doi:10.1104/pp.110.161273. 20921160 PMC2948990

[49] White TJ, Bruns T, Lee S, Taylor J. Amplification and direct sequencing of fungal ribosomal RNA genes for phylogenetics. In: Innis MA, Gelfand DH, Sninsky JJ, White TJ editors. PCR protocols: a guide to methods and applications. Vol. 38. San Diego (CA (USA)): Academic Press; 1990. p. 315–322.

[50] Al-Taweel K, Iwaki T, Yabuta Y, Shigeoka S, Murata N, Wadano A. A bacterial transgene for catalase protects translation of D1 protein during exposure of salt-stressed tobacco leaves to strong light. Plant Physiol. 2007. 145(1):258–265. doi:10.1104/pp.107.101733 PMC1976566 17660354

[51] Manzo D, Ferriello F, Puopolo G, Zoina A, D’Esposito D, Tardella L, Ferrarini A, Ercolano MR. *Fusarium oxysporum* f. sp. *radicis-lycopersici* induces distinct transcriptome reprogramming in resistant and susceptible isogenic tomato lines. BMC Plant Biol. 2016;16:53. doi:10.1186/s12870-016-0740-5. 26920134 PMC4769521

[52] Moghaieb REA, Ahmed DS, Abdelhadi AA, Sharaf AN. An efficient and reproducible regeneration and transformation protocol in tomato (*Solanum lycopersicum* L.). Australian Journal of Basic and Applied Sciences. 2015;9:411–416.

[53] Rogers SO, Bendich AJ. Extraction of DNA from milligram amounts of fresh, herbarium and mummified plant tissues. Plant Mol Biol. 1985;5(2):69–76. doi:10.1007/BF00020088. 24306565

[54] Sambrook J, Fritschi EF, Maniatis T. Molecular cloning: a laboratory manual. New York (USA): Cold Spring Harbor Laboratory Press; 1989.

[55] Chakraborty N, Chandra S, Acharya K. Biochemical basis of improvement of defense in tomato plant against Fusarium wilt by CaCl_2_. Physiol Mol Biol Plants. 2017;23(3):581–596. doi:10.1007/s12298-017-0450-y. 28878497 PMC5567711

[56] Aebi HE. Catalase. Bergmeyer HU editor. Methods of enzymatic analysis. Weinhem (Germany): verlag Chemie. 1983. 273–286.

[57] Onsa GH, Bin Saari N, Selamat J, Bakar J. Purification and characterization of membrane-bound peroxidases from *Metroxylon sagu*. Food Chem. 2004;85(3):365–376. doi:10.1016/j.foodchem.2003.07.013.

[58] Kumar A, Dutt S, Bagler G, Ahuja PS, Kumar S. Engineering a thermo-stable superoxide dismutase functional at subzero to >50°C, which also tolerates autoclaving. Sci Rep. 2012;2:387. doi:10.1038/srep00387. 22548128 PMC3339387

[59] Shapiro SS, Wilk MB. Analysis of variance test for normality (complete samples). Biometrika. 1965;52(3/4):591–611. doi:10.2307/2333709.

